# Examining the Association between Life-Space Mobility and Cognitive Function in Older Adults: A Systematic Review

**DOI:** 10.1155/2019/3923574

**Published:** 2019-06-02

**Authors:** Nileththi A. De Silva, Michael A. Gregory, Shree S. Venkateshan, Chris P. Verschoor, Ayse Kuspinar

**Affiliations:** ^1^Department of Interdisciplinary Sciences, McMaster University, Hamilton, ON, Canada L8S 4L8; ^2^School of Rehabilitation Science, McMaster University, Hamilton, ON, Canada L8S 1C7; ^3^Department of Psychology, Neuroscience and Behavior, McMaster University, Hamilton, ON, Canada L8S 4L8; ^4^Department of Health Research Methods, Evidence, and Impact, McMaster University, Hamilton, ON, Canada

## Abstract

**Purpose:**

The purpose of this review is to investigate the relationship between life-space mobility and cognition in older adults.

**Methods:**

MEDLINE, Embase, CINAHL, and PsycINFO were searched through December 2018 for studies containing measures of life-space mobility and cognitive function. Two independent reviewers screened studies. Eligible studies were combined using a random-effects model, and heterogeneity was assessed using the *I* ^2^.

**Results:**

Thirty-five articles were identified for review. A moderate and statistically significant association (pooled *r* = 0.30, 95% confidence interval 0.19 to 0.40.) was observed between life-space mobility and cognition among nine studies. Life-space mobility demonstrated small-to-moderate associations with domain-specific cognitive functioning, particularly executive function, learning, memory, and processing speed. Furthermore, individuals who had restricted life-space mobility (Life-Space Assessment ≤ 40) experienced a steeper decline in cognition (*β* = 0.56 and *p* = 0.0471) compared to those who did not (Life-Space Assessment ≥ 41).

**Conclusion:**

This review examined the association between life-space mobility and cognitive function in older adults. The results suggest that a moderate relationship between life-space mobility and cognition exists, whether adjusted or unadjusted for covariates such as sociodemographics, mental health, functional capacity, and comorbidities.

## 1. Introduction

Mobility is an integrated motion referring to forms of movement ranging from the use of assistive devices to public transport [1]. Mobility loss in aging manifests in several ways, including the development of gait impairments or changes in driving behaviour. Mobility has been conceptualized using a variety of measures. For instance, the spatiotemporal assessment of gait is commonly used for the evaluation of mobility impairments in dementia populations [2]. Within rehabilitation research, other scales and measures of mobility, such as the Elderly Mobility Scale [3] and Performance-Oriented Mobility Assessment [4], are used. Although these measures have been validated, they fail to acknowledge life-space mobility (LSM) which describes the spatial environment a person moves through within a specified time period [5]. LSM not only captures social interactions of individuals; as larger life spaces can reveal higher levels of community engagement [6], it can also be representative of real-world applications of functional skills, which traditional, performance-based mobility tests do not consider.

Another characteristic of aging is the decline in cognitive function, which encompasses executive function, learning, memory, attention, visual-spatial ability, and perceptual-motor function. Cognitive impairment is differentially distributed throughout the aging population [7]. In fact, less than 40% of those with mild cognitive impairment have been reported to progress to dementia and Alzheimer's disease [8]. These findings suggest that since the degree of cognitive degeneration varies from person to person, preventative actions [9, 10] may deter the onset of cognitive impairment, and in turn, conversion to dementia, which is an irreversible condition.

The concurrent decline in mobility and cognitive function in aging suggests an association between the two variables [11, 12], while the lack of uniformity seen in the decline presents an area for active research.

Therefore, the aim of this systematic review is to summarize the associations between LSM and cognition (global and specific domains) in older adults.

## 2. Methods

### 2.1. Search Strategy

A systematic literature search of 4 electronic databases Ovid MEDLINE (1946 to December 2018), Embase (1974 to December 2018), CINAHL (1982 to December 2018), and PsycINFO (1806 to December 2018) was conducted. Please refer to Supplementary Table 1 for a summary of the databases included and the search terms used for this review.

### 2.2. Study Selection

#### 2.2.1. Study Design

Randomized controlled trials, retrospective and prospective cohort studies, case series, case-control studies, and cross-sectional studies were eligible for inclusion.

#### 2.2.2. Participants

Studies that sampled community-dwelling or nursing/long-term care residents, over the age of 65, exhibiting a range of mobility limitations and cognitive conditions, were considered.

#### 2.2.3. Outcomes of Interest

Studies were considered for inclusion if they met the following criteria: (1) contained an outcome measure for LSM and cognition and (2) reported a quantitative association between LSM and cognition.

#### 2.2.4. Inclusion/Exclusion Criteria

Articles were included if they (1) included older adults over the age of 65 years, (2) measured LSM and cognition using a standard outcome measure (e.g., Life-Space Assessment and Mini-Mental State Examination), and (3) reported a quantitative association between LSM and cognition. Manuscripts were excluded if they were (1) protocol papers; (2) review articles; (3) unpublished studies, conference abstracts, or dissertations; (4) non-peer-reviewed articles; (5) book chapters; (6) manuscripts published outside of the English language, and (7) manuscripts that did not use a questionnaire to assess LSM (i.e., GPS technologies).

### 2.3. Data Extraction and Analysis

Title and abstract screening (Level 1) was performed by two independent reviewers (NAD and SV). Both reviewers (NAD and SV) performed the full-text review and data extraction for the final inclusions. Disagreements between the two reviewers during Level 1 screening were resolved through discussion [13]. Data extraction was performed using a prepared data extraction sheet, which included (1) study and population characteristics; (2) cognitive outcomes utilized; (3) life-space mobility outcome utilized; and (4) the associated results. The linear relationships and explained variances were interpreted according to Cohen's guidelines, where an *r* value of 0.10 indicates a small effect, *r* value of 0.30 indicates a medium effect, and an *r* value of 0.50 indicates a large effect and *R* ^2^ value of 0.02 indicates a small effect, *R* ^2^ of 0.13 indicates a medium effect, and *R* ^2^ of 0.26 indicates a large effect [14].

A forest plot was constructed by MedCalc Statistical Software version 18.6 [15] to combine the correlation coefficient values across applicable studies. For the purpose of this forest plot, weighted averages of *r* values were taken if a study presented more than one correlation value for the association between LSM and cognition [16]. The *I* ^2^ statistic was used to assess heterogeneity, which is the percentage of total variation across studies that is due to between-study differences rather than chance [17]. The *I* ^2^ ranges between 0 and 100%, with higher values indicating greater heterogeneity [17]. A *p* value of <0.05 and an *I* ^2^ value >50% indicated significant heterogeneity [17].

### 2.4. Study Quality Assessment

The SIGN 50 checklist as shown in Supplementary Table 3 was used to evaluate the quality of the included citations [18]. The SIGN 50 evaluates internal validity across 20-items that fall within four domains. The quality of each study was ranked based on the maximum attained score: (1) 0–50% (low quality), (2) 51–74% (moderate quality), and (3) ≥75% (high quality). Two reviewers (MG and NAD) performed quality assessments, and any disagreements were addressed through discussion [18]. A third party (SV) adjudicated any persisting disagreements [18] to reach a consensus on quality scores, which are presented in Supplementary Table 4.

## 3. Results

### 3.1. Study Descriptions

**Figure 1:**
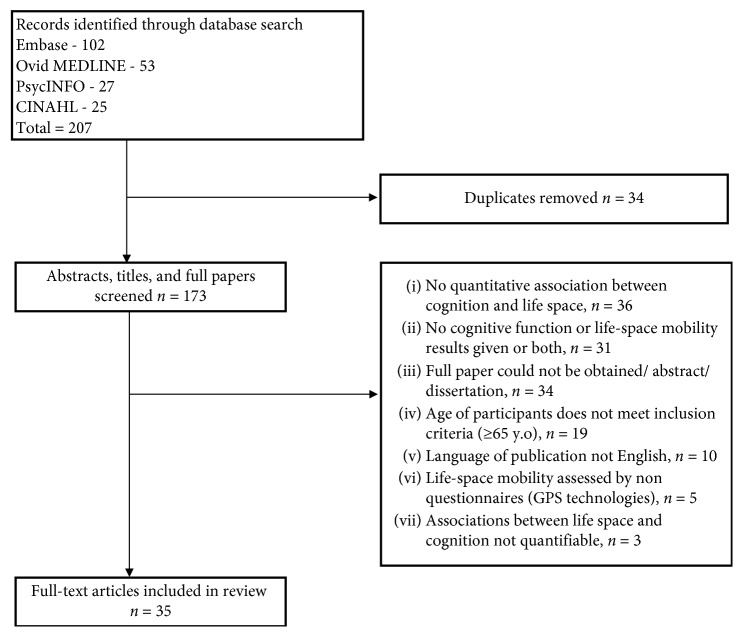
Flow diagram to illustrate study selection.

In total, 207 papers were exported from the databases and 173 abstracts were reviewed after exclusion of duplicates. Thirty-five were included in this review as illustrated in Figure 1. Supplementary Table 4 summarizes the characterizations of the included studies. Of the thirty-five papers, 21 studies employed a cross-sectional design [19–39], 13 studies used a longitudinal/prospective design [40–52], and 1 study [53] employed a randomized control trial design. There were 17 studies [19, 27, 29–31, 34, 37–39, 41–43, 49–53], whose primary objective concerned cognition, and 18 studies [20–26, 28, 32, 33, 35, 36, 40, 44–48] which assessed cognition but did not identify cognition as a variable of primary interest. The sample sizes ranged from 20 to 3892.

An age limit of ≥65 was applied to the electronic database searches; however, two studies with an inclusion criteria for age <65 surfaced from the search. Bergland et al. [22] included participants ≥64, while Mortenson et al. [25] included participants ≥60. The two studies were deemed sufficient for inclusion as the mean ages of their respective samples exceeded 80 years of age. Among the 35 studies included in the review, the mean age of participants ranged from 65 to 87.6.

Twenty-six studies [21–27, 31, 33–43, 46–51, 53] in the review had a predominately female sample (>50% females). Fifteen studies [19, 24, 26, 28–31, 33, 35, 37, 48, 50–53] included participants whose average years of education were less than 13, while six studies [27, 41–45] had a population whose mean years of education exceeded 13 years. Nine studies [21, 23, 26, 27, 41–43, 45, 46] had a primarily Caucasian population (>50% Caucasian vs other races), three studies [24, 28, 50] assessed a Hispanic or Latin population (Mexican, Colombian, and Brazilian), and 5 studies [29–32, 53] evaluated an East Asian sample (Chinese and Japanese). While the majority of the studies included a sample of community-dwelling older adults, three studies [22, 25, 35] exclusively assessed subjects residing in long-term care and nursing homes.

Twenty-six percent of studies [21, 27–29, 32, 43, 44, 50, 53] included individuals with healthy cognitive function (i.e., Mini-Mental State Examination (MMSE) score >25 and Leganés cognitive test (LCT) scores >22) [54]. Of the remaining studies, 6 [22, 25, 34, 38, 39, 46] included a mild to moderate (i.e., MMSE 17–26) [55] and severe cognitive impairment (MMSE scores <24 [56] and Modified Mini-Mental State (3MS) Examination scores <77/78 [57]) and 4 studies [35, 40, 45, 46] included dementia patients.

### 3.2. Assessment of Study Quality

The quality assessment results as measured by the SIGN 50 checklist [58] show that 21 studies [19–23, 25–29, 31, 37–39, 41, 44–48, 53] were of moderate quality (i.e., 51–74%), ranging from 57.9% to 73.3%, and 14 studies [24, 30, 32–36, 40, 42, 43, 49–52] were of high quality (i.e., ≥75%), ranging from 75.0% to 86.7%.

### 3.3. Outcome Measures Used in Studies

A summary of the cognitive tests used in this review are shown in Supplementary Table 2. Global cognitive functioning (i.e., evaluated via the MMSE or Montreal Cognitive Assessment (MoCA)) was most commonly assessed (*n* = 27/34 studies) [19–26, 29–34, 36, 38, 39, 41–44, 47–52]. Domain-specific cognitive functions, including attention, executive function, learning, memory, language, processing speed, perceptual speed, and visuospatial abilities, were evaluated in twelve of the included studies [21, 27, 29–32, 37, 40, 43, 49, 51, 53].

The life-space measures' descriptions and frequencies can be found in Table 1. Within the included citations, 27 [19, 20, 24–26, 28–34, 36–41, 45–53] used the University of Alabama at Birmingham Life-Space Assessment (LSA), 3 studies employed a modified Life-Space Assessment for cognitively impaired patients [34, 38, 39], 5 studies [21, 27, 42–44] administered the Life-Space Questionnaire (LSQ), 3 [21, 43, 44] used a modified LSQ, and 3 [22, 25, 35] used the Nursing Home Life-Space Diameter (NHLSD).

### 3.4. Association between LSM and Cognition

**Figure 2:**
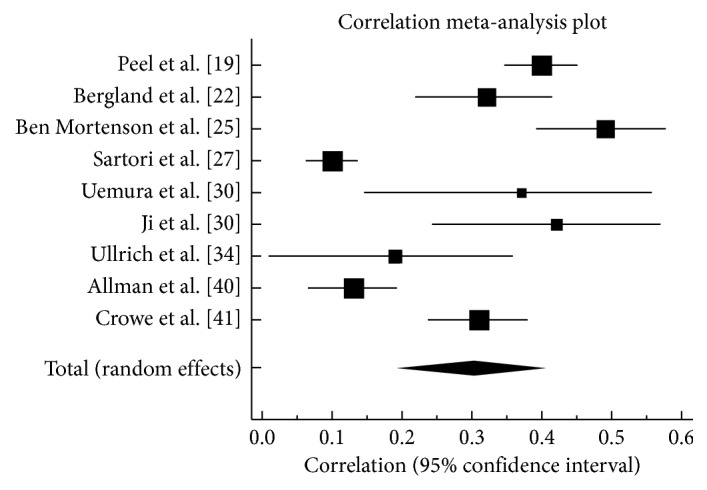
Forest plot with correlation coefficient values (*r*) for the association between life-space mobility and cognition.

Only nine studies [19, 22, 25, 27, 30, 32, 34, 40, 41] assessed the correlation between LSM and cognition. As shown in Figure 2, the random-effect pooled correlation was 0.30 (95% CI 0.19 to 0.40) (Cohen's medium effect size), which is significant as the confidence interval excludes zero. High heterogeneity (*I* ^2^ = 93.9%) was found across the nine studies.

### 3.5. Summary of Cognitive Domain Results

The correlation coefficients obtained for the relationship between learning and memory and LSM ranged from 0.22 to 0.23 [27, 32] (Cohen's small effect size) and −0.19 to 0.37 [27, 30] perceptual speed/processing speed/visuospatial processing speed (Cohen's small-to-medium effect size). The correlation coefficients obtained for the relationship between executive function and LSM ranged from 0.13 to 0.26 [27, 40] (Cohen's small effect size). Furthermore, for executive function, longitudinal analyses demonstrated that poor performance on the Trail Making Tests (i.e., ≥240 seconds to complete or ≥4 mistakes) significantly predicted lower life-space mobility scores (*β* = *−*11.03, *p*=0.006) [51] while good performance (i.e., <60 seconds) was found to predict higher life-space scores (*β* = 3.81, *p*=0.012) [49].

Certain studies [19, 20, 24, 27, 34, 35, 41, 43] assessed the relationship between LSM and cognition using multiple regression analyses. These regression models included variables such as sociodemographics, mental health, general health, fear of falling, instrumental activities of daily living, physical activity/performance, transportation difficulty, social activities, living situation, comorbidity, and vascular risk factors.  Table 2 presents the numerical associations for a mixed sample of older adults harbouring a range of cognitive conditions. The *R* ^2^ values for the association between LSM and cognitive function in these studies ranged from 0.11 to 0.59 [19, 20, 27, 30, 34, 35, 41] (Cohen's small to large effect size).

### 3.6. LSM Category Results

As shown in Table 3, cognitive function results varied by the level of LSM. When comparing cognition between those restricted to the household and those who regularly travel beyond the town, it is apparent that the prevalence of cognitive impairment is much higher among older adults with lower life space. Longitudinal analyses [43, 47, 50] found that individuals who are restricted to the home versus those who are not have a higher risk of developing mild cognitive impairment (HR = 1.17 (95% CI 1.06 to 1.28)) [43] and Alzheimer's disease (HR = 1.21 (95% CI 1.08 to 1.36)) [43] and accelerated decline in global cognitive function [50]. Global cognition was also significantly higher for participants with maintained and early-decline LSM (life space declined >10 points but remained stable after the 2nd follow-up) versus those with constricted or late-decline LSM (life space remained stable at the 1st follow-up then decreased >10 points by the 2nd follow-up) (*p*=0.023) [48]. The majority of the studies included in the review suggest that constricted LSM may be associated with cognitive impairment. However, in a recent cohort study involving community-dwelling older adults, decline in LSM was not observed to significantly depend on a change in global cognition over a period of two years [52].

Cross-sectional studies found restricted life space (i.e., LSM <60) at baseline was associated with lower global cognition compared to unrestricted life space (*p*=0.002, *p* < 0.001) [33, 44]. In separate cross-sectional studies, an increase in cognition was found to reduce the odds of being restricted to the home (OR = 0.40 (95% CI 0.23 to 0.69)) [21] and neighbourhood (OR = 0.97 (95% CI 0.87 to 1.07); OR = 0.40 (95% CI 0.21 to 0.75)) [21, 28]. However, contrary to these studies, a recent cross-sectional study reported that older adults who travelled out of the home on a daily basis did not have significantly higher global cognition (*p*=0.502) [36].

### 3.7. Association between LSM and Cognition in Individuals with Mild to Moderate Cognitive Impairment

Six studies [30, 31, 34, 38, 39, 42] included participants with known mild to moderate cognitive impairment at baseline as diagnosed by a physician, determined by a psychometric algorithm, meeting Peterson's internationally accepted criteria [62] or having MMSE scores between 17 and 26 [63]. In these studies, amnestic mild cognitive impairment was not significantly associated with life space. Longitudinally, having amnestic and nonamnestic mild cognitive impairment was found to have negative effects on driving frequency, but not on life space over a five year period as measured by the Life-Space Questionnaire [42]. Likewise, further analyses showed that life space did not significantly vary between controls and those with mild cognitive impairment in a cross-sectional study (*p*=0.128) [31]. Studies that performed sensitivity analyses to identify a cutoff value between low and high LSM found that cognitively impaired subjects have a substantially lower value (LSA = 26.8) [38] compared to samples of cognitively healthier adults (LSA = 52.3) [47].

### 3.8. Association between LSM and Cognition in Individuals with Dementia

Four studies [35, 40, 45, 46] targeted individuals with dementia. The collective results of the studies were largely in agreement with one another; as life space increased, the proportion of men and women with dementia decreased (*p* < 0.001) [45, 46]. While nursing home residents with dementia had a significantly wider LSM than subjects without (*p*=0.003), healthy cognitively functioning older residents exhibited more dependency on others or equipment to achieve mobility compared to the cognitively impaired residents (*p*=0.013) [35].

## 4. Discussion

The goal of this review was to discern the relative association between LSM and cognitive function in older adults. The key results reported herein suggest that a moderate relationship between LSM and cognition exists, whether or not adjusted for covariates such as sociodemographics, mental health, functional capacity, and comorbidities. Longitudinal studies suggest that restriction to the house and areas immediately proximate to the household (i.e., equivalent to an LSA score ≤40 and LSQ <4) increases the risk for developing cognitive impairment in good cognitively functioning seniors.

The pooled association across nine studies was moderate in magnitude and statistically significant. However, this value must be interpreted with caution as the heterogeneity (*I* ^2^) was substantially large (93.9%). The high heterogeneity may be attributed to variations in population characteristics such as the inclusion of dementia and the different LSM and cognitive measures used in the studies.

Previous reviews have described the relationship between mobility and cognition; however, they quantified mobility within the realm of physiological and biomechanical functions and omitted the consideration of mobility with respect to life-space utilization across diverse populations. For example, Demnitz et al. [64] conducted a systematic review of cross-sectional studies analysing cognition and mobility among healthy older adults and discovered that features of mobility including gait, lower extremity function, and balance, yielded small effect sizes for their association with cognitive function.

Similarly, Clouston et al. [65] conducted a systematic review of physical and cognitive function changes in older cohorts and found that physical functioning as defined by measures like grip strength and walking speed were significantly associated with changes in cognitive function. Our study extends these findings to the broader involvement of the spatiotemporal environment, which can only be captured by measures of LSM. For example, when low physical fitness and cognitive function limit the ability to ambulate, acquire information, or make decisions, the attainment of higher life spaces also become hindered, further depriving older adults of enriching auditory, visual, and tactile stimuli. Therefore, it is important to consider environmental fluctuations (i.e., transition from home to nursing home, hospitalization, or driving cessation) that occur in the course of aging in tandem with mobility and cognitive declines.

The findings of the current review also found that mobility outcomes were not uniformly linked with all cognitive domains as was also revealed by Demnitz et al. [64] and Clouston et al. [65]. For example, when considering specific cognitive domains, the review found that processing speed presented the largest range of small–to-moderate mixed associations with LSM, while executive function and learning and memory showed small positive associations with LSM. Global cognitive function also exhibited a moderate association with LSM. Additionally, processing speed played a more significant role in determining life space among amnestic mild cognitively impaired older adults [30] while reasoning emerged as a strong predictor of life space among subjects with healthy cognition [27].

### 4.1. Limitations

The majority of the studies contained a high proportion of Caucasians and females, limiting the generalizability of our results and consideration of the influence of cultural and racial differences on the LSM and cognition relationship. Also, many of the studies measured cognitive function using the MMSE, which has an underrepresentation of executive function with the majority of the scoring items evaluating orientation and language [66]. Thus, despite the MMSE being regarded as a tool to screen for cognitive impairment, its capacity to test global cognitive function is limited and may not be suitable to assess change in cognition over time, as was done in seven of the included studies [19, 23, 24, 34, 41, 49, 50].

### 4.2. Implications

The findings of this review expand on the theoretical connections between environmental factors and cognitive function. As postulated by the Environmental Complexity Theory, exposure to cognitive stimulation in diverse and heterogeneous spaces during the earlier decades of aging may reinforce the cognitive reserves of executive function, learning, memory, and processing speed, which subsequently contributes to the effective utilization of mobility in later life [67]. This proposition may explain why weakened global cognition increases the likelihood of restricted LSM, which then increases the risk of dementia. The Scaffolding Theory of Aging and Cognition similarly proposes that external lifestyle influences may contribute to the brain's neural and functional capacity to adapt and compensate for age-related changes [68]. Some of the external interventions advocated by the authors Reuter-Lorenz and Park to boost the brain's compensatory mechanisms include exercise, intellectual engagement, social activity, and cognitive training [68]. The findings of this review suggest that higher life spaces provide opportunities for intellectual and social engagement, thereby facilitating the compensatory scaffolding of the brain to impose a protective barrier against cognitive decline.

This review also demonstrated that most nursing and long-term care residents possess substantially lower LSM and cognition compared to community-dwelling older adults. Cognitive and mobility impairments are common reasons for admission into long-term care or nursing homes [69, 70]. However, exposure to heterogeneous spaces within these institutions is particularly limited as there are fewer opportunities for activity engagement [71]. Jansen et al.'s study of movement within nursing homes found that while meal times were associated with higher transits out of the room, residents remain largely confined to their rooms during their free time [72]. Therefore, there is a need to incorporate social and physical activities into institutional schedules that encourage excursions outside of the room, unit, and facility for older adults.

This study did not delineate the temporal ordering of mobility and cognitive decline; however, it suggests that both LSM and cognition can play predictive roles in the trajectory of these declines. While it is unclear whether or not LSM restrictions precede cognitive dysfunction, it is important to recognise that LSM may be a more discernible outcome to measure compared to cognitive function, due to the observability of the contributing factors to life space such as the frequency and duration of movement as well as size of social networks. Thus, LSM may be a more practical initial target for early aging interventions. Furthermore, research on the combined effects of cognitive training and life-space enhancing activities such as building relationships outside of the home through explorative community engagements is yet to be explored.

## Figures and Tables

**Table 1:** Summary of life-space mobility measures.

Life-space mobility test	No. of questions	Administration time frame	Criteria	Scoring	Maximal score
UAB Life-Space Assessment (UAB LSA) [19, 20, 24–26, 28–33, 36, 37, 40, 41, 45–51, 53] Life-Space Assessment adapted for cognitive impairment (LSA-CI) [34, 38, 52]	15	LSA: last 4 weeks LSA-CI: past week	Life space zones [59]: (i) Bedroom (ii) Outside bedroom (iii) Outside the home (iv) Neighbourhood (v) Beyond neighbourhood Frequency: (i) Less than once/week (ii) 1–3 times/week (iii) 4–6 times/week (iv) Daily Assistance required [59]: (i) Person (ii) Device	(i) Life-space zone scored 1–5 (ii) Frequency scored 1–4 (iii) Assistance scored 1-2 [59]	120
Life space Questionnaire [21, 27, 42–44]	9	Last 3 days	Life space zones: [60] (i) Bedroom (ii) Area immediately outside the home (iii) Area outside of home (iv) Areas in immediate neighbourhood (v) Area outside of neighbourhood (vi) Areas outside of county/city (vii) Area outside of state/province (viii) Area outside of country	(i) “Yes” responses scored 1 (ii) “No” responses scored 0 [60]	(i) 9 (ii) Modified LSQ out of 6
Nursing home life-space diameter [22, 25, 35]	9	Past 2 weeks (can be completed by nursing home staff) [61]	Life space diameters: [61] (i) Diameter 1: bedroom (ii) Diameter 2: outside the room (iii) Diameter 3: outside the unit (iv) Diameter 4: outside the facility Frequency [61]: (i) 5, >3 times/day (ii) 4, 1–3 times/day (iii) 3, >2 times/day (iv) 2, weekly (v) 1, less than weekly (vi) 0, never Assistance (optional) [61]: (i) Human assistance	(i) 1 (diameter 1 × frequency 1) (ii) 2 (diameter 2 × frequency 2) (iii) 3 (diameter 3 × frequency 3) (iv) 4 (diameter 4 × frequency 4) (v) Score × 2, if independent mobility, done without human assistance [61]	100

**Table 2:** Associations between life-space mobility and cognition.

Cognitive domain/assessment	*R* ^2^ cognition as exposure	*R* ^2^ LS as exposure
Clinical dementia rating scale [35]	0.12–0.14 (*R* ^2^ change not available)	No data
Executive function [27]	0.12 (*R* ^2^ change 0.009)	No data
Learning and memory [27]	0.12 (*R* ^2^ change 0.006)	No data
Perceptual motor/processing speed/visuospatial ability [27, 30]	0.11 (*R* ^2^ change 0.03) to 0.37	No data
Mini-Mental State Examination (MMSE) [19, 20, 34, 41]	0.42 to 0.59 (*R* ^2^ change 0.07)	0.14

*R* ^2^ values are derived from multiple regression models including other covariates. The increment change in *R* ^2^ after cognition added to the multiple regression model is reported in brackets.

**Table 3:** Cognitive results based on life-space categorizations.

Scores	Life-space category: restricted to house and household vicinity			Life-space category: neighbourhood level	Life-space category: out of town
	Modified LSQ < 1	LSA ≤ 40	LSA ≤ 52.3	LSA 41–60	LSA ≥ 61
Results	Older adults in this category were at a higher risk of developing MCI compared to individuals who travel beyond the town (HR = 1.17 (95% CI 1.06 to 1.28)) [43]	Older adults in this category had a higher rate of cognitive decline over 5 years (*β* = 0.56, *p*=0.0471) compared to people who visited the neighbourhood and beyond (LSA ≥ 41) [50]	Older adults in this category had lower cognitive function than those with higher scores (LSA > 52.3) (*p* < 0.001) [47]	Older adults in this category had lower rate of global cognitive decline (*β* = 0.85, *p*=0.0026) over 5 years compared to those who were constricted to their home (LSA ≤ 40) [50]	Older adults in this category had lowest rate of global cognitive decline over 5 years (*β* = 1.03, *p*=0.0004) compared to those with neighbourhood life space (LSA 41–60) [50]
	Older adults in this category were at a higher risk of developing AD compared to individuals who travel beyond the town (HR = 1.21 (95% CI 1.08 to 1.36)) [43]				

## Supplementary Materials

Supplementary Materials Supplementary Table 1: Ovid MEDLINE search terms modified for Embase, CINAHL, and PsycINFO (from November to December 2017). Supplementary Table 2: summary of cognitive tests administered in studies. Supplementary Table 3: SIGN 50 adapted for prospective, randomized controlled trial, and cross-sectional studies. Supplementary Table 4: descriptive information for each study included in the systematic review.
