# Supplementary material for: Examining the Association between Life-Space Mobility and Cognitive Function in Older Adults: A Systematic Review
Source: J Aging Res. 2019 Jun 2;2019:3923574. doi: 10.1155/2019/3923574 (PMC6589294; doi:10.1155/2019/3923574)
Supplement: Supplementary Materials — Supplementary Table 1: Ovid MEDLINE search terms modified for Embase, CINAHL, and PsycINFO (from November to December 2017). Supplementary Table 2: summary of cognitive tests administered in studies. Supplementary Table 3: SIGN 50 adapted for prospective, randomized controlled trial, and cross-sectional studies. Supplementary Table 4: descriptive information for each study included in the systematic review. [file 3923574.f1.docx]

Supplemental Table 1:

OVID MEDLINE search terms modified for EMBASE, CINAHL and PSYCHINFO (from November to December 2018)

| Search Steps | Search Terms |
| --- | --- |
| 1 | exp Cognition*/ or cognition.mp. |
| 2 | cognitive domain*.mp. |
| 3 | exp memory/ or exp learning, episodic/ or long-term/ or exp memory, short-term/ or mental recall/ or exp spatial memory |
| 4 | processing speed.mp. |
| 5 | social cognition.mp. |
| 6 | visuospatial skill*.mp. or visuospatial process*.mp. |
| 7 | exp mental processes/ or exp executive function/ or exp thinking/ |
| 8 | cognitive dysfunction.mp. or exp Cognitive Dysfunction/or dementia/ or neurocognitive disorder*/ or Alzheimer’s disease/ or cognitive impair*.mp. |
| 9 | life space mobility.mp. |
| 10 | life space assessment*.mp. |
| 11 | life space questionnaire*.mp. |
| 12 | life space diary.mp. |
| 13 | Nursing Home Life space Diameter.mp. |
| 14 | life space restriction.mp. |
| 15 | life space.mp. |
| 16 | 1 or 2 or 3 or 4 or 5 or 6 or 7 or 8 |
| 17 | 9 or 10 or 11 or 12 or 13 or 14 or 15 |
| 18 | 16 and 17 |
| 19 | Limit to 65 and over |

Supplemental Table 2: Summary of cognitive tests administered in studies

| Cognitive Test | Cognitive Function Criteria |
| --- | --- |
| The Mini Mental State Examination-MMSE [19–26,29–34,36,38,39,41–44,47–51] | - Global cognitive function |
| Trail Making Test [30,37,49,51] | - Executive function - Processing speed |
| Wechsler Memory Scale [21,30,43] | - Memory |
| Verbal Fluency Test / Word Fluency Test [21,29,37] | - Executive functioning - Language |
| Digit Span test [21,37,43] | - Working Memory |
| The Montreal Cognitive Assessment [31,37,52] | - Global cognitive function |
| The Teng Modified Mini-Mental State- 3MS [45,46] | - Global cognitive function |
| Number Comparison [21,43] | - Visuospatial ability |
| Progressive Matrices [21,43] | - Visuospatial ability |
| Hopkins Verbal Learning Test [27,42] | - Learning & memory |
| Rey Auditory Verbal Learning Test [27,42] | - Learning & memory |
| Useful Field of View [27,42] | - Processing speed |
| 5 Cog Test [31,53] | - Attention - Memory - Visuospatial - Language - Reasoning – executive function |
| Word Series, Letter Series and letter sets test [27,42] | - Reasoning – executive function |
| Paragraph recall subtest of the Rivermead Behavioral Memory Test [27,42] | - Recall - Memory |
| Word list memory recall and recognition [21,43] | - Memory |
| Immediate and delayed recall of East Boston story [21,43] | - Episodic Memory |
| Boston Naming Test [21,43] | - Semantic Memory |
| Symbol digit modalities [21,43] | - Perceptual speed |
| Judgement of Line Orientation Test [21,43] | - Visuospatial ability |
| Stroop Test [37,43] | - Perceptual speed |
| Wechsler Adult Intelligence Scale II/III digit symbol coding and symbol search [30,37] | - Processing speed |
| Self-efficacy Questionnaire[40] | - Social Cognition |
| Clock Drawing Test – Clox [40] | - Executive function |
| Free and Cued Reminding Test [37] | - Episodic Memory |
| Leganés cognitive test [28] | - Temporal, spatial orientation - Semantic memory - Immediate memory - Late memory – recall - Logical memory |
| Alzheimer’s Disease Scale [30] | - Memory - Orientation - Language |
| The Clinical Dementia Rating Scale Sum of Boxes [35] | - Memory - Orientation - Executive function |
| Reading Test [21] | - Working Memory |

Supplemental Table 3:

SIGN 50 adapted for Prospective, Randomized Controlled Trial and Cross-sectional Studies

Note: that a retrospective study (i.e. database or chart study) cannot be rated higher than +.

| Study identification (*Include author, title, year of publication, journal title, pages*) | | | | |
| --- | --- | --- | --- | --- |
| SECTION 1: INTERNAL VALIDITY | | | | |
| *In a well conducted cohort study:* | Does this study do it? | | | |
| 1.1 The study addresses an appropriate and clearly focused question. | Yes | No | Unclear | NA |
| SELECTION OF SUBJECTS | | | | |
| 1.2 The two groups being studied are selected from source population that are comparable in all respects other than the factors under investigation. | Yes | No | Unclear | NA |
| 1.3 The study indicates how many of the people asked to take part did so, in each of the groups being studied. | Yes | No | Unclear | NA |
| 1.4 Are the criterion used to define cognitive function/status clearly stated? | Yes | No | Unclear | NA |
| 1.5 Evidence from other sources was used to demonstrate the validity and reliability of the methods used to determine cognitive function/status? | Yes | No | Unclear | NA |
| 1.6 The likelihood that some eligible participants might have the outcome (cognitive impairment) at the time of enrollment is assessed and taken into account in the analysis? | Yes | No | Unclear | NA |
| 1.7 Was an adequate randomization allocation concealment method used? Not applicable for cross-sectional/retrospective studies. *Not applicable to cross-sectional/retrospective studies* | Yes | No | Unclear | NA |
| 1.8 Were participants and investigators kept blind to group allocation? *Not applicable to cross-sectional/retrospective studies* | Yes | No | Unclear | NA |
| 1.9 Was the percentage of individuals or cluster recruits that dropped out from either arm/group of the study before the end of the study reported? | Yes | No | Unclear | NA |
| 1.10 Comparison was made between participants who completed the study and those who dropped out. | Yes | No | Unclear | NA |
| 1.11 The outcomes (mobility) are clearly defined? | Yes | No | Unclear | NA |
| 1.12 The assessment of the outcome was made blind to cognitive status/group membership? | Yes | No | Unclear | NA |
| 1.13Where blinding was not possible, is there some recognition that knowledge of a participants cognitive status could have influenced mobility-related data collection/the outcome? | Yes | No | Unclear | NA |
| 1.14 Are the selected outcome measures (life space mobility) measured in a valid way? | Yes | No | Unclear | NA |
| 1.15 Were the selected outcome measures (life space mobility) measured in a valid way? | Yes | No | Unclear | NA |
| 1.16 Was cognition and/or life Space Mobility assessed more than once? | Yes | No | Unclear | NA |
| 1.17 For multi-site studies, were comparisons made to determine similar results between sites? | Yes | No | Unclear | NA |
| CONFOUNDING | | | | |
| 1.18 The main potential confounders are identified and taken into account in the design and analysis | Yes | No | Unclear |  |
| STATISTICAL ANALYSIS | | | | |
| 1.19 Were confidence intervals, or some other form of variance in the outcome measures provided? | Yes | No |  |  |
| 1.20 Were power analyses relating to the primary outcome performed? | Yes | No |  |  |
| SECTION 2: OVERALL ASSESSMENT OF THE STUDY | | | | |
| 2.1 Were attempts to minimize the risk of bias or confounding evident? | High quality: | | | |
|  | Moderate Quality: | | | |
|  | Low Quality: | | | |
| 2.2 Taking into account clinical considerations, the evaluation of the methods used, and the statistical power/approach of the study, does there appear to be clear evidence of an association between exposure (cognitive function) and outcome (life space mobility) or vice versa ? | Yes | No | Unclear |  |
| 2.3 Are the results of the study directly applicable to the patient group under investigation? | Yes | No | Unclear |  |
| 2.4 Notes. Summarise the author’s conclusion. Add any comments on your own assessment of the study, and the extent to which it answers your questions and mention any areas of uncertainty raised above. | | | | |

Supplemental Table 4: Descriptive information for each study included in the systematic review

| First Author (year)  Country  Study Design | Participants | Purpose | Cognition Outcome | Life-Space Mobility Outcome | Results | SIGN50 Score |
| --- | --- | --- | --- | --- | --- | --- |
| Allman (2004) [40]   - USA - Prospective (1.5 years) | Community-dwelling older adults (N=905)  African American (50%; n=452)   - 48% between 65-74 years - 51% female, 35% <7^th^ grade education   Caucasian (50%; n=453)   - 55% between 65-74 years - 51% female, 9% <7^th^ grade education | To define racial similarities and differences in mobility, and to identify predictors of mobility change | *Executive Function*  CLOX <10  African Americans:  214 (47%)  Caucasians:  88 (19%) | LSA  African Americans:  57.0 (24.5)  Caucasians:  72.7 (22.6) | African Americans had lower baseline LS mobility, and this disparity was accompanied by racial differences in SES and health  Despite racial disparities in LS mobility, EF was negatively associated with LS mobility for African Americans (r= -0.13) and Caucasians (r= -0.14)  Dementia was retained as a significant predictor of LS mobility decline for African Americans only [standardized β = - 0.07] | 75.0%  High |
| Peel (2005) [19]   - U.S.A - Cross-sectional | Community-dwelling older adults  (n=998)   - 75.3 (6.7) years, 50% female - 50% African American, 27% >high school education | Examine the relationship between LS mobility, socio-demographics, physical function, affect, and cognition | *Global Cognition*  MMSE:  25.0 (4.8) | LSA:  64.1 (24.9) | Global cognitive functioning is negatively associated with LSA (r=-0.40)  MMSE, affect, basic-ADL, instrumental-ADL, physical function, and demographics explained 58.9% of the variance in LSA scores; however, MMSE scores did not independently contribute to the explained variance in LSA (β=-0.066) | 64.7%  Moderate |
| Allman (2006) [20]   - USA - Cross-sectional | Community dwelling older adults (n=1000)  Caucasian (n=500)   - 74.7 (no SD) years, 50% female - 6% < 6^th^ grade education   African Americans (n=500)   - 75.9 (no SD) years, 50% female - 35% < 6^th^ grade education | To understand racial differences in mobility among older adults | *Global Cognition*  MMSE  Caucasians:  27.1 (no SD)  African Americans:  23.0 (no SD) | LSA  Caucasians:  70.9 (no SD)  African Americans:  56.5 (no SD) | Global cognitive functioning was independently associated with LS mobility (β= 0.159); however, this association was strongest for rural, female African Americans (β = 0.29)  Global cognitive functioning was independently associated with LS mobility among urban-dwelling Caucasians (β = 0.227) and African American men (β = 0.203)  Sociodemographic factors, health and mental status explained 49% of LS variance(R^2^=0.49) | 76.5%  High |
| Barnes (2007) [21]   - USA - Cross-sectional | Community-dwelling older adults  (n=837)   - 80.9 (7.1) years, 75% female - 91% Caucasian, 14.4 (3.1) years of education | To examine the properties of a modified version of the Life Space Questionnaire | *Global Cognition*  MMSE:  27.9 (2.1)  *Global Cognitive Function*  Composite score derived from domain-specific composites | LSQ-Modified (not reported) | Global cognitive functioning is related to LS mobility    For every unit increase in global cognition, there was an approximate 50% reduction in the likelihood of reporting the smallest LS:   - Homebound:  [OR=0.40 (95% CI 0.23 to 0.69)] - Within Neighborhood: [OR=0.40 (95% CI 0.21 to 0.75)] - Outside Neighborhood: [OR=0.61 (95% CI 0.40 to 0.96)] | 70.6%  Moderate |
| Crowe (2008) [41]   - USA - Longitudinal (4 years) | Community dwelling adults (n=624)   - 73.8 (5.9) years, 53% female - 51% Caucasian, 44% < high school education | Is greater LS associated with reduced cognitive decline over 4 years | *Global Cognition*  MMSE:  26.1 (3.9) | LSA:  69.8 (22.8) | Global cognitive function was associated with LS (r=0.31)  LS contributed to the prediction of cognitive decline over 4 years [β= -0.156, Adjusted R^2^=.14], even after excluding of those with low baseline cognitive function (i.e., MMSE < 21) | 75.0%  High |
| Bergland (2010) [22]   - Scandinavia - Cross-sectional | Nursing home residents in need of daily assistance (n=322)   - 85 (7.7) years, 74% female - Education and ethnicity not specified | Investigate the associations between physical function, cognition, mobility and independence in nursing home residents | *Global Cognition*  MMSE–Swedish: 18.1 (7.9) | NHLSD:  41.0 (23.0) | LS mobility [(i.e., NHLSD area (r=0.23) and dependence (r=0.32)] is positively associated with global cognitive functioning | 60.0% Moderate |
| O’Connor (2010) [42]   - USA - Prospective (5 years) | Community dwelling older adults with and without MCI (N=2355)  Amnestic MCI (aMCI; n=82)   - 76.8 (6.5) years, 51% female - 72% Caucasian, 13.1 (3.2) years of education   Non-amnestic MCI (naMCI; n=140)   - 75.8 (6.1) years, 81% female - 54% Caucasian, 12.5 (2.4) years of education   Multi-domain (md-MCI; n=82)   - 78.9 (6.0) years, 52% female - 54% Caucasian, 11.7 (2.7) years of education   HC (n=2051)   - 72.6 (5.3) years, 75% female - 78% Caucasian, 14.0 (2.6) years of education | Examine trajectories of changes in mobility amongst older drivers who are cognitively healthy, and those with psychometrically defined MCI, aMCI, naMCI, and md-MCI | *Global Cognition*  MMSE (>23)  MCI classifications psychometrically determined using composite scores from a variety of cognitive tests | LSQ (baseline)  aMCI: 6.96 (1.3)  naMCI: 6.77 (1.2)  md-MCI: 6.95 (1.4)  HC: 7.31 (1.2) | Compared to the HC, naMCI and md-MCI participants reported lower baseline LSQ scores  A negative main effect of MCI group membership on driving space was revealed  Although MCI group membership was associated with reductions in driving frequency and changes in driving behaviour, no significant relationship between MCI and the LSQ were identified | 81.0%  High |
| James (2011) [43]   - USA - Prospective (8 years) | Community dwelling older adults (N=1294)  Did not develop AD (n=1114)   - 77.5 (7.6) years; 75% female - 68% Caucasian, 14.6(3.3) years of education   Developed AD (n=180)   - 84.1 (6.3) years; 70% female - 89% Caucasian, 14.3(3.0) years of education | Determine the association between constricted LS mobility and the risk for incident dementia, MCI, or rapid cognitive decline in older adults | *Global cognition*  MMSE  Did not develop AD: 28.2 (1.8)  Developed AD:  26.4 (2.8)  *Incident cognitive impairment:*  Dementia: NINCDS-ADRD criteria  MCI: cognitive impairment that did not meet dementia criteria | LSQ-Modified  Did not develop AD:  0.55 (1.1)  Developed AD:  0.99 (1.4) | LS was associated with baseline cognitive function and the trajectory of cognitive decline over time  After adjustment for covariates, those who developed AD had more constricted LS at baseline; AD risk increased 21% for each successively smaller zone of LS [HR=1.21 (95% CI 1.08 to 1.36)]  The association between constricted LS and incident AD was higher for African American [HR=1.49 (95% CI 1.06 to 2.08)] than Caucasians [HR=1.19 (95% CI 1.05 to 1.34)]  A more constricted life space was associated with an increased risk of MCI [HR = 1.17 (95% CI 1.06 to 1.28)]; persons constricted to their home environment were 1.6 time more likely to develop MCI than those with the largest LS | 82.4%  High |
| Popescu (2011) [23]   - Canada - Cross-sectional | Ophthalmology clinic patients  (N=272)   - 97% Caucasian - Education and ethnicity not specified   AMD (n=68)   - 82.6 (5.8) years, 76% female   FCD (n=49)   - 79.4 (7.3) years, 84% female   GC (n=82)   - 76.5 (7.4) years, 87% female   HC (n=73)   - 72.8 (4.6) years, 62% female | Examine the extent of mobility limitations among ophthalmology patients compared to older adults with healthy vision | *Global Cognition*  MMSE-blind version  AMD: 18.8 (2.8)  FCD: 19.5 (2.5)  GC: 19.0 (3.0)  HC: 20.7 (1.4) | LSA  AMD: 37.5 (17.3)  FCD:45.4 (24.6)  GC: 54.0 (25.5)  HC: 73.8 (18.5) | Compared to HC, ophthalmology patients displayed worse global cognitive functioning and lower LS mobility  Global cognitive functioning contributes to the explained variance in LSA among ophthalmology patients [β=1.2 (95% CI: 0.19 to 2.21)] | 66.7%  moderate |
| Maki (2012) [53]   - Japan - RCT | Community dwelling older adults  (N=150)   - Ethnicity not specified   Intervention (n=75)   - 71.9 (4.1) years, 69% female - 11.8 (2.5) years of education   Control (n=75)   - 72.0 (3.9) years, 72% female - 11.9 (2.3) years of education | To evaluate the efficacy of a municipality‐led walking program under the Japanese public Long‐Term Care Insurance Act to prevent mental decline | *Global Cognition*  MMSE  Intervention: 27.7 (1.9)  Control: 27.9 (2.0)  *Domain-specific Cognition*  Learning & Memory  Executive function  Language  Visuospatial abilities  Sustained attention | LSA  Intervention: 94.5 (16.6)  Control: 90.4 (20.0) | Although improvements in executive functioning (i.e., word fluency) and LS mobility were observed following the intervention, no between-group differences were revealed | 77.3%  Moderate |
| Makizako (2013) [29]   - Japan - Cross-sectional | Healthy, independent community-dwelling older adults (N=20)   - 76.1 (6.7) years, 50% female - 10.7 (2.6) years of education, ethnicity not specified   Outdoors daily (n=7)  Outdoors non-daily (n=13) | To investigate the relationship between daily outdoor travel and prefrontal cortex  activation during execution of the VFT using NIRS. | *Global Cognition*  MMSE  Total: 26.1 (2.6)  Outdoors daily: 27.0 (2.8)  Outdoors non-daily: 25.6 (2.5)  *Executive Function*  VFT (not reported) | LSA sub-questionnaire (not reported) | No between-group differences in global cognitive function or EF were identified  Compared to non-daily outdoor travellers, prefrontal cortical oxygenation during VFT was greater for those who travel outdoors daily:   - right inferior front gyrus:  daily: 0.262 mM/mm vs. non-daily: 0.086 mM/mm (p=0.03) - left inferior front gyrus: daily: 0.276 mM/mm vs. non-daily: 0.093 mM/mm (p = 0.04) | 73.3%  Moderate |
| Mortensen (2012) [25]   - Canada - Cross-sectional | LTC residents using wheelchairs  (N=264)   - 84.2 (8.6) years, 69% female - 38% using proxy-respondents - Education and ethnicity not specified | Explore the relationship between wheelchair-related factors, mobility, and participation among LTC residents | *Global Cognition*  MMSE: 15.5 (10.7) | NHLSD: 41.1 (19.2) | Global cognitive functioning is positively associated with NHLSD score (r=0.49)  Path analysis suggests that this indirect relationship is mediated by wheelchair skills, and depression | 68.4%  moderate |
| Popescu (2012) [26]   - Canada - Cross-sectional | Ophthalmology clinic patients  (N=315)   - 97% Caucasian   AMD (n=81)   - 82.4 (5.9) years, 75% female - 9.2 (3.3) years of education   FCD (n=55)   - 79.1 (7.2) years, 84% female - 11.0 (4.4) years of education   GC (n=91)   - 76.4 (7.6) years, 57% female - 10.7 (4.3) years of education   HC (n=88)   - 73.1 (4.5) years, 59% female - 11.6 (3.9) years of education | To examine whether patients with age-related eye diseases are more likely to show signs of depression compared HC, and to determine whether reduced mobility mediates this relationship | *Global Cognition*  MMSE–blind  AMD: 18.8 (2.8)  FCD: 19.5 (2.5)  GC: 19.4 (2.5)  HC: 20.7 (1.4) | LSA  AMD: 38.1 (17.7)  FCD: 46.2 (24.1)  GC: 54.4 (25.7)  HC: 73.3 (19.7) | Older adults with age-related eye diseases performed worse on measures of cognition, ADL, and LS mobility, and were more likely to experience participation restrictions due to FOF. | 72.2%  Moderate |
| Sartori (2012) [27]   - USA - Cross-sectional | Community-dwelling older adults (N=2737)   - 73.6 (5.9) years, 76% female - 73% Caucasian, 13.5(2.7) years of education | To examine the relationship between cognitive function and LS, determine whether performance-based tests of everyday function are associated with life space, and understand how this relationship is impacted by personal control beliefs | *Global Cognition*  MMSE: 27.3 (2.0)  *Memory Composite*  0 (2.5)  *Reasoning Composite*  0 (2.7)  *Processing Speed Composite*  0 (2.5) | LSQ  7.2 (1.3) | LS mobility was positively associated with global cognition (r= 0.17) and processing speed (r = -0.185), and positively associated with memory (r = 0.206) and reasoning (r = 0.259)  In separate models, memory (β = 0.089, R^2^= -0.116), reasoning (β = 0.117, R^2^= -0.119), and processing speed (β = -0.067, R^2^= -0.113) were significant predictors of LS mobility    Reasoning provided the greatest predictive utility for LS mobility | 68.8%  Moderate |
| Shah (2012) [44]   - USA - Longitudinal (4+ years) | Community-dwelling older adults without dementia (N=571)   - Ethnicity not specified   LS Maintained (n=268)   - 76.1 (7.4) years, 46% female - 15.2 (3.1) years of education   LS Constricted (n=303)   - 80.6 (6.9) years, 54% female - 14.3 (2.9) years of education | To explore whether being licensed to drive is associated with maintaining spatial movement in older persons initially reporting maximum spatial mobility | *Global Cognition*  MMSE  LS Maintained:  28.4 (1.8)  LS Constricted:  27.9 (2.1) | LSQ-Modified  (not reported) | 303 participants with maximum spatial mobility at baseline reported subsequent LS constriction during follow-up; at baseline, these individuals were older, had lower global cognitive function, slower gait speed, greater social isolation, and were less likely to be licensed to drive  62% of those reporting incident LS constriction experienced recovery of spatial mobility during follow-up; a valid driver’s license mediated this observation [HR=2.0 (95% CI: 1.27 to 3.17)] | 66.7%  Moderate |
| Snih (2012) [24]   - USA - Cross-sectional | Community dwelling Mexican Americans (N=728)   - 84.2 (4.2) years, 65% female - 100% Hispanic, 89% < high school education | To examine the factors associated with life‐space mobility in older Mexican Americans | *Global Cognition*  Cognitive impairment (i.e., MMSE <21): 33%  Cognitive Impairment  Yes: 37.8 (19.6)  No: 44.8 (19.8) | LSA  Total: 41.7 (20.9) | No differences in LS mobility at baseline between those with and without cognitive impairment  Once adjusted for applicable covariates, global cognition was not retained as a significant predictor of LS mobility [β = - 0.30 (SE 1.54)] | 76.5%  High |
| Curcio (2013) [28]   - Brazil & Colombia - Cross-sectional | Community dwelling adults (N=300)   - Female prevalence and ethnicity not specified   Brazil (n=150)   - 69.6 (3.0) years - 6.5 (4.6) years of education   Colombia (n=150)   - 69.1 (6.4) years - 4.8 (3.5) years of education | To assess the reliability, construct and convergent validity of the LSA in Latin American older populations | *Global Cognition*  LCT  Brazil: 28.4 (2.6)  Colombia: 25.9 (3.5) | LSA- Spanish  Brazil: 59.6 (17.8)  Colombia: 51.9 (19.0) | Unadjusted bivariate regression identified cognitive function as a predictor of LSA in Latin Americans (β=1.08)  In adjusted binary logistic regression analyses, higher cognition was associated with lower odds of being restricted to neighbourhood.  LS-maximal ≤ 3 [OR=0.97 (95% CI 0.87 to 1.07)] ; LS-Independent ≤ 3 [OR=0.94 (95% CI 0.86 to 1.01) scores | 70.6%  Moderate |
| Uemura (2013) [30]   - Japan - Cross-sectional | Community-dwelling older adults with aMCI (N=69)   - 75.4 (6.9) years, 56.5% males - 11.0 (2.6) years of education, ethnicity not specified | To identify factors associated with LS mobility in older adults with aMCI | *Global Cognition*  MMSE: 26.8 (1.8)  ADAS-Cog: 6.4 (2.2)  *Executive Function*  TMT-B:  204.5 (109.1) sec  *Processing Speed*  DSC: 45.7 (14.7) | LSA – Japanese  96.3 (19.9) | LS mobility was positively associated with processing speed (r=0.365), but not EF (r=-0.21) or global cognition (r=-0.07)  Stepwise multiple regression analyses retained processing speed as a significant predictor of LS mobility (β = 0.278)  DSC, FOF and IADL explained a large amount of LS variance (R^2^=0.37) | 86.7%  High |
| Mackey (2014) [45]   - USA - Prospective (4 years) | Community dwelling older men (N=3892)   - 77.8 (4.5) to 85.6 (6.5) years - 90% Caucasian, 57% ≥ college education | To evaluate the relationship between LS mobility and mortality in older men | *Global Cognition*  Teng MMSE (range): 80.7 (12.3) to 93.5 (6.2) | LSA  84.9 (24.2) | Baseline cognitive functioning was positively associated with LS mobility; dementia prevalence was negatively associated with LS mobility  Compared to men with the highest LSA scores, those scoring ≤ 40 on the LSA were at increased risk of non-cancer mortality [HR=2.15 (95% CI: 1.12 to 4.11] | 60.0%  Moderate |
| Maki (2014) [31]   - Japan - Cross-sectional | Community dwelling older adults with and without MCI (N=157)   - Ethnicity not specified   MCI (n=37)   - 73.1 (4.4) years, 54% female - 11.5 (3.0) years of education   HC (n=120)   - 71.9 (4.1) years, 75% female - 11.9 (2.2) years of education | To evaluate the impact of subjective memory complaints on QOL in those with and without MCI | *Global Cognition*  MMSE  MCI: 25.7 (1.9)  HC: 28.4 (1.5)  MoCA memory: MCI: 8.8 (3.5)  HC: 15.2 (4.5)  MoCA EF: MCI: 14.7 (7.2)  HC: 21.9 (6.4) | LSA  MCI: 96.3 (17.4)  HC: 90.8 (19.7) | LS mobility did not differ between HC and MCI group  Although between-group differences in objective cognitive functioning were apparent, no differences in reports of subjective memory complaints were observed | 61.1%  Moderate |
| Ji (2015) [32]   - China - Cross-sectional | Community-dwelling older adults (N=100)   - 72.2 (5.1) years, 50% female - Ethnicity and Education not specified | To examine the reliability and validity of the Chinese version of the LSA | *Global Cognition*  MMSE: 25.5 (3.4) | LSA-Chinese  71.9 (20.2) | Global cognition was positively correlated with LS mobility (r= 0.42)  Of the individual items on the MMSE, temporal association and drawing (both r=0.31) were most strongly associated with LS mobility | 86.7%  High |
| Rantakokko (2015) [33]   - Finland - Cross-sectional | Community-dwelling older adults  (N=848)   - Ethnicity not specified   Restricted LS (LSA <60; n=348)   - 82.4 (4.1) years, 75% female - 8.7 (3.6) years of education   Unrestricted LS (LSA ≥60; n=500)   - 79.4 (3.9) years, 53% female - 10.2 (4.4) years of education | Examine the association between perceived environmental barriers to and facilitators for outdoor mobility with life-space among older people | *Global Cognition*  MMSE  Restricted LS:  25.7 (3.1)  Non-restricted LS: 26.5 (2.4) | LSA (not reported) | Those with restricted LS were older, more likely female, had a greater number of chronic conditions, lower education, and lower cognitive functioning  Those reporting ≥2 environmental mobility barrier were 1.85 time more likely to have restricted LS [OR: 1.85 (95% CI: 1.24-2.78)]  Those reporting 4-7 mobility facilitators were approximately half as likely to have restricted LS [OR: 0.64 (95% CI: 0.42-0.96)] | 81.3%  High |
| Mackey (2016) [46]   - USA - Prospective (5 years) | Community dwelling older women (N=1498)   - 87.6 (3.4) years - 88% Caucasian, 38% ≥ college education | To evaluate the relation between LS mobility and mortality in older women | *Global Cognition*  Teng MMSE (range):  76.1 (14.8) to 91.1 (8.6) | LSA  55.4 (23.6) | Baseline cognitive functioning was positively associated with LS mobility  Dementia prevalence was negatively associated with LS mobility increased | 60.0%  Moderate |
| Portegijs (2016) [47]   - Finland - Longitudinal (2 years) | Community dwelling adults (baseline N=848, follow-up N=755)   - Median age of 80.4 (IQR 7.4) - 62% female - Ethnicity and education not specified | Investigate the association between LS mobility and ADL disability status to define cut-off scores for baseline LSA performance and LSA change over time | *Global Cognition*  MMSE [(median, (IQR)]  Baseline LSA≤52.3:  26.0 (4.0)  Baseline LSA>52.3:  27.0 (3.0) | LSA [median, (IQR)]  64.0 (30.4) | An LSA cut-off score of 52.3 provided 86% sensitivity and 74% specificity in identifying those who would develop ADL limitations during follow-up   - These individuals were older, had more chronic conditions, and lower physical and cognitive function   A decline of >11.7 in LSA over 2-years provided 76% sensitivity and 71% specificity in identifying those who develop ADL limitations during follow-up | 57.9%  Moderate |
| Rantakokko (2016) [48]   - Finland - Prospective (2 year) | Community-dwelling older adults  (N=848)   - Ethnicity not specified   Maintained LS (stable; n=335)   - 80.4 (4.1) years, 66% female - 9.6 (3.9) years of education   Late Declined LS (2^nd^ year; n=178)   - 80.3 (4.1) years, 62% female - 9.4 (3.8) years of education   Early Declined LS (1^st^ year; n=211)   - 80.6 (4.3) years, 60% female - 10.0 (4.8) years of education   Constant Declined LS (both; n=27)   - 79.9 (3.7) years, 59% female - 10.6 (4.8) years of education | Examine the interrelationship between changes in LS mobility and QoL over 2 years | *Global Cognition*  MMSE (baseline)  Maintained LS:  26.5 (2.6)  Late Declined LS:  25.8 (2.6)  Early Declined LS:  26.6 (2.6)  Constant Declined LS:  26.1 (3.0) | LSA  (baseline;2-year)  Maintained LS:  58.9 (19.6); 66.0 (22.1)  Late Declined LS:  64.4 (17.5); 55.2 (19.1)  Early Declined LS:  73.5 (19.3); 61.5 (22.0)  Constant Declined LS:  83.9 (19.8); 42.3 (22.9) | Mean 2-year change in LSA score was -3.8 (16.6) points (range -82 to +54)  Those experiencing Late Decline LS had lowest baseline cognitive functioning  QoL scores decreased across all groups over 2-years  After adjusting for covariates, participants demonstrating Late Decline (β=4.13) or Early Decline (β=3.58) in LS were most likely to report reduced QoL | 70.0%  Moderate |
| Poletti (2017) [37]   - France - Cross-sectional | Community-dwelling right-handed older adults (N=21)   - Ethnicity not specified   Healthy older adults (n=11)   - 73.6 (6.4) years, 69% female - 13.0 (2.7) years of education   Cognitively impaired adults (n=10)   - 81.7 (9.0) years, 80% female - 7.6 (3.2) years of education | To investigate how healthy older adults and cognitively impaired adults differ in performing Fitts’ aiming task | G*lobal Cognition* MoCA  HOA: 28.6 (1.2)  CIP: 24.2 (3.5)  *Processing Speed*  Digit Symbol Coding  HOA: 64.6 (11.2) CIP: 37.2 (8.6)  Symbol Search  HOA: 28.1 (5.0) CIP: 16.9 (3.8)  *Episodic Memory*  Immediate Free Recall:  HOA: 35.8 (5.9)  CIP: 25.4 (7.1)  Delayed Free Recall:  HOA: 13.8 (1.5) CIP: 10.5 (3.6)  *Working Memory*  Digit Span Forward  HOA: 11.3 (1.6)  CIP: 8.5 (1.6)  *Digit Span Backward*  HOA: 8.3 (2.0)  CIP: 6.0 (1.1)  *Verbal Fluency*  Category Fluency HOA: 24.4 (5.2) CIP: 15.7 (4.6)  Letter Fluency  HOA: 17.6 (2.7) CIP: 11.5 (2.6)  *Inhibition*  Victoria Stroop Test HOA: 1.2 (0.4)  CIP: 2.3 (1.5)  *Cognitive Flexibility*  TMT  HOA: 16.9 (10.1) CIP: 74.7 (41.6) | LSA (not reported) | Healthy older adults’ LSM was 96.2 (17.0) was significantly higher than cognitively older patients’ LSM was measured to be 46.2 (17.3) | 61.1%  Moderate |
| Portegijs (2017) [36]   - Finland - Cross-sectional | Community-dwelling older adult (N=848)   - 80.6 (4.3) years, 64% female - Education and ethnicity not reported | To investigate association between objectively recorded and perceived entrance-related environmental barriers for mobility and the whether objectively recorded and perceived barriers were associated with moving out of home daily for those with and without lower extremity limitations. | *Global Cognition*  MMSE Moving out of home daily: 27.0 (IQR 3.0)  Moving out home less than daily: 27 (IQR 4.0) | LSA (not reported) | MMSE scored between participants who move out of the home daily for the last 4 weeks and those who do not were not significant | 75.0%  High |
| Poranen-clark (2018a) [49]   - Finland - Longitudinal (2 years) | Community-dwelling older adults (N=169)   - 82.2 (4.1) years, 59% female - Education and ethnicity not reported | To investigate the temporal associations between EF and life-space mobility among community-dwelling older people | *Global Cognition*  MMSE baseline: 26.3 (2.7)  follow-up: 26.2 (3.0)  *Executive Function*  Delta TM < Good baseline: 58%  follow-up: 63% | LSA  baseline: 63.1 (19.7)  follow-up: 61.5 (21.2) | Higher baseline global cognitive functioning [β =1.22 (95% CI: 0.29 to 2.15)] and EF [unstandardized path coefficient 3.81 (95% CI: 0.84 to 6.78)] were significant predictors of LS mobility at follow-up  Baseline LS mobility did not predict cognitive functioning at follow-up [β=0.01 (95% CI: -0.01 to 0.03)] | 78.9%  High |
| Silberschmidt, (2017) [50]   - USA - Longitudinal (5 years) | Community-dwelling Mexican Americans without dementia (N=432)   - 83.8 (3.9) years, 66% female - 5.8 (4.0) years of education, 100% Hispanic | To examine the association between LS mobility and cognitive decline over 5 years | *Global Cognition*  MMSE: 25.7 (3.2) | LSA  44.6 (20.7) | The adjusted rate of decline for total MMSE was 1.48 per year  Compared to those in the lowest LS mobility category (i.e., 0-20), participants with the highest LS mobility (≥61) experienced slower rates of cognitive decline over 5 years (β=1.03) | 75.0%  High |
| Béland (2018) [52]   - Canada - Longitudinal (2 years) | Community-dwelling older adults (N=1643)   - 67.4% of participants >75 years of age and 32.6% of participants between 65-74 years of age, 49.8% female - 10.7 (not reported) years of education - Ethnicity not reported | To examine the longitudinal association between cognitive decline and change in LSM in community-dwelling older adults, and to investigate the impact of moderators and mediators on this association | *Global Cognition*  MoCA  baseline: 23.9 (4.0)  follow-up 1: 24.5 (4.0)  follow-up 2: 24.3 (4.0) | LSA  baseline: 62.6 (24.9)  follow-up 1: 60.6 (24.1)  follow-up 2: 61.1 (24.5) | Intercept of LSA on intercept of MoCA was significant [β =0.168, 95% CI 0.12 to 0.22]  Slope of LSA on intercept and slope of MoCA did not reach significance.  Gait and depression were found to mediate association between cognition and LSM while grip strength was found to moderate the relationship between LSM and cognition | 81.3%  High |
| Poranen-Clark (2018b) [51]   - Finland - Longitudinal (2 years) | Community dwelling older adults  (N=157)   - Ethnicity not specified     Poor EF (n=54)   - 84.1 (4.0) years, 65% female - 7.9 (3.4) years of education   Intermediate EF (n=50)   - 82.2 (4.1) years, 60% female - 9.8 (4.8) years of education   Good EF (n=53)   - 81.5 (4.2) years, 59% female - 11.3 (4.0) years of education | Investigate association between EF and LS mobility, and determine whether perceived walking difficulties, lower extremity performance, and transportation difficulties underlie this relationship | *Global Cognition*  MMSE: 26.2 (2.6)  *Executive Function*  Delta TMT  Poor EF: incomplete tests and/or >4 errors  Intermediate EF: 95-179 sec  Good EF: ≥94 sec | LSA  Poor EF: 50.3 (20.6)  Intermediate EF: 62.9 (18.2)  Good EF: 64.2 (21.2) | A positive association between EF and LS mobility was observed at baseline  In the fully adjusted model this interaction was not significant for those with Intermediate [β= 0.39 95% CI −5.57 to 6.35] or Poor EF [β =-4.57, 95% CI −11.03 to 1.89]  Association between LS and EF attenuated by functional status and transportation difficulties | 82.4%  High |
| Svedrup (2018) [35]   - Norway - Cross-sectional | Nursing home residents (N=696)   - 84.4 (7.5) years, 56% female - 8.0 (IQR 2.0) years of education - Ethnicity not specified | To describe mobility at admission to nursing homes and to assess the association between mobility and dementia | *Dementia Assessment*  CDR: 10.3 (4.3) | NHLSD area: 22.0 (IQR 17.0)  NHLSD dependency: 36.0 (IQR 26.0) | NHLSD area and NHLSD dependency scores significantly different among dementia subgroups  In multiple regression analyses, adjusted for age, gender, marital status, GMHR, CDR-SOB significantly predicted NHLSD dependency [β =-0.53, 95% CI -0.94 to -0.12] but not NHLSD area  Socio-demographics, GMHR and CDR-SOB explained NHLSD area (R^2^=0.12) and NHLSD dependency (R^2^=0.14), however cognition only contributed 1% of LSM’s explained variance  In planned group comparisons, subjects with severe dementia has significantly worse NHLSD area than those with moderate dementia  Those with mild dementia has significantly higher NHLSD dependency scored than subjects without dementia.  Those with moderate dementia had significantly higher NHLSD dependency scores than those with severe dementia and subjects with severe dementia had significantly lower NHLSD area than those with moderate dementia | 76.5%  High |
| Ullrich (2018a) [34]   - Germany - Cross-sectional | Community-dwelling older adults with mild to moderate cognitive impairment (N=118)   - 82.3 (6.0) years, 76% female - 31.4% of subjects has school, 50% had vocational school and 18.6% had university | To describe the LSM in older patients with mild to moderate CI after geriatric rehabilitation | *Global Cognition*  MMSE: 23.3 (2.4) | LSA-CI composite  23.9 (13.2)  LSA-CI Maximal  3.7(1.2)  LSA-CI -E  2.5 (1.2)  LSA-CI -I  1.1(1.4) | MMSE and LSA-CI composite score significantly and positively correlated (r=0.19)  MMSE did not significantly predict LSA-CI.  Activity behaviour, cognition, SPBB, social activities, falls efficacy scale, fear of falling avoidance, living situation, gender, age, private unpaid care explained a large amount of variance (R^2^= 0.42) | 81.3%  High |
| Ullrich (2018b) [38]   - Germany - Cross-sectional | Community-dwelling older adults with mild to moderate cognitive impairment (N=118)   - 82.3 (6.0) years, 76% female - Education and ethnicity not reported | To determine cut-off for LSA-CI to differentiate between low and high LSM for older adults with cognitive impairment | *Global Cognition*  MMSE: 23.3 (2.4) | LSA-CI  23.9 (13.2) | MMSE scores for those with low LSM (<26.8) were significantly lower than for subjects with high LSM (≥ 26.8) | 58.8%  Moderate |
| Ullrich (2018c) [39]   - Germany - Cross-sectional | Community-dwelling older adults with mild to moderate cognitive impairment (N=118)   - 82.3 (6.0) years, 76% female - 31.4% of subjects had school only,68.6% of subjects had vocational or academic studies | To investigate the validity, reliability, sensitivity to change, and feasibility of a modified University of Alabama at Birmingham Study of Aging Life-Space Assessment (UAB-LSA) in older persons with cognitive impairment (CI) | *Global Cognition*  MMSE: 23.3 (2.4) | LSA  Not reported | LSA-CI-E and MMSE positively and significantly correlated (r=0.21)  Other LSA-CI measures such as composite, maximal and independent life space scores did not correlate significantly with MMSE. | 72.2%  Moderate |
| ***Note:*** *All data reported as mean (SD), unless specified otherwise. All prevalence and frequency data were rounded to the nearest percentage.*  ***Cognitive Outcomes:*** CLOX has a maximum score of 15, where higher scores reflect better performance; DSC score reflects number of correct responses in 2 minutes, where higher scores represent better performance; LCT has a maximums score of 32, where higher scores reflect better performance; 3MS has a maximum score of 100, where higher scores reflect better cognitive functioning; MMSE and MoCA have a maximum score of 30, blind MMSE has maximum score of 18, where higher scores reflect better cognitive functioning; TMT is scored in seconds, where higher scores reflect poorer performance; Symbol Search score represents number of correct responses adjusted for errors in 2 minutes, where higher scores reflect better performance; CDR scores range from 0 to 3 where a score of 1 or more indicates dementia and CDR-SOB scores range from 0 to 18 where higher scores indicate more cognitive impairment. ***Mobility Outcomes:*** LSA has a maximum score of 120, where higher scores reflect greater mobility. LS-Maximal is the highest LS attained with help from person or equipment while LS-Independent is the highest LS attained without help from a person or equipment; LSQ has a maximum score of 9, where higher scores reflect greater mobility; LSQ-Modified (maximum score of 6) was reverse coded, where lower scores reflect greater life space mobility; NHLSD scores range from 0-100, where higher scores reflect greater life space mobility; LSA-CI is an adaptation of LSA for cognitively impaired individuals with a maximal score of 120, where higher scores reflect greater mobility; LSA-CI-E reflects maximum LS attained with help of equipment.  ***Abbreviations:*** 95% CI, 95% confidence interval; AD, Alzheimer’s disease; ADL, activities of daily living; AMD, age-related macular degeneration; CLOX, Clock Drawing Test; DSC, digit symbol coding test; EF, executive function; GC, glaucoma; FCD, Fuchs’ corneal dystrophy; HC, healthy controls; LTC, long-term care; FOF, Fear of Falling; NINCDS-ADRD, National Institute of Neurological and Communicative Disorders and Stroke and the Alzheimer Disease and Related Disorders; LCT, Legane’s Cognitive Test; LS, life space; LSA, Life Space Assessment; LSQ, Life Spare Questionnaire; MCI, mild cognitive impairment; MMSE, Mini-Mental State Examination; NHLSD, Nursing Home Life Space Diameter Assessment; QoL, quality of life; RCT, randomized controlled trial; sec, seconds; SE, standard error; SES, socioeconomic status; VFT, verbal fluency task; LSA-CI, Life Space Assessment-Cognitive Impairment; GMHR, General Medical Health Rating; CDR-SOB, Clinical Dementia Rating-Sum of Boxes. | | | | | | |
